# Quality improvement of saliva by chewing tapioca pearls in bubble tea drinks: a randomized experimental trial

**DOI:** 10.12688/f1000research.28028.1

**Published:** 2021-02-01

**Authors:** Juni Handajani, Dinda Kusumajati, Hania Fathiyah, Heni Susilowati, Regina T.C. Tandelilin

**Affiliations:** 1 Department of Oral Biology, Faculty of Dentistry, Universitas Gadjah Mada, Yogyakarta, Indonesia, 55281, Indonesia; 2 Dental Hygiene Study Program, Faculty of Dentistry, Universitas Gadjah Mada, Yogyakarta, Indonesia, 55281, Indonesia

**Keywords:** bubble tea, salivary C Reactive Protein (CRP), salivary calcium (Ca)

## Abstract

**Background:** Bubble tea drinks contain tea and tapioca pearls. Chewing tapioca pearls in bubble tea drinks may increase salivary components. Because of its proteins, inorganic components, and enzymes, saliva plays an important role in the body’s defense against bacteria and viruses. This study aims to analyze the effect of chewing tapioca pearls in bubble tea drinks on salivary C-reactive protein (CRP) and calcium (Ca) levels.

**Methods:** The inclusion criterion was 18–25 years of age. The exclusion criteria were receiving medication, using dentures, a history of dry mouth, smoking and systemic disease. In the first week of the experiment, subjects drank bubble tea with tapioca pearls for three days (intervention week). In the second week, the same subjects drank tea without pearls for three days (control week). Each subject drank the bubble tea for 5 minutes per day over 3 days. Saliva samples were collected on the first day before bubble tea consumption (pretest) and on the third day after tea consumption (posttest). Saliva collection was performed in the morning (09:00 am–12:00 pm) for 1 minute. Sixty saliva samples were collected from 15 subjects. Salivary CRP levels were measured using a commercial ELISA kit, and Ca levels were determined using semi-quantitative test strips.

**Results:** Salivary CRP decreased significantly on the third day in the intervention group but showed no significant difference with the control group. Calcium levels increased significantly on the third day in both groups.

**Conclusion:** Bubble tea drinks could improve the quality of saliva by decreasing salivary CRP and increasing Ca levels.

**Trial registration: **ClinicalTrials.gov, NCT04670341 (17 ^th^ December 2020).

## Introduction

The oral cavity is the main gateway for microorganisms to the body. Saliva, which is secreted from the salivary glands, is a body fluid that is important for maintaining a healthy oral cavity and body health. Saliva was recently determined to be a very important route through which COVID-19 may be spread. Saliva is an essential component of the body’s defense because it contains large amounts of proteins, inorganic components, and enzymes. Saliva has been developed as a fluid for supporting diagnostics, i.e detection antibodies for HIV, and quantitation of steroid hormone, salivary cortisol, and neuropeptide for biomarkers for psychological research ^1^.

Saliva contains proteins and peptides with antibacterial, antiviral, and antifungal activities. Proteins found in saliva include histatin, statherin, alpha and beta defensins, and C-reactive protein (CRP). Histatins 1, 3, and 5 are histatin derivates known to inhibit the growth of *Candida albicans*. The main function of statherin is to inhibit the crystallization of phosphate from saliva and the growth of anaerobic bacteria ^2^. Defensins function as antibacterial and antiviral compounds ^3^. CRP is a marker of inflammation. Under normal circumstances, CRP levels in humans are very low; in acute inflammation, however, CRP levels may increase by several hundred times the normal level. CRP levels in saliva are believed to originate from blood circulation to the salivary glands through passive diffusion ^4^.

The main inorganic components of saliva, namely, Ca, phosphates, and bicarbonates, are involved in tooth protection, especially tooth remineralization and demineralization. High levels of Ca and phosphate in saliva also affect the maturation and remineralization of teeth ^5, 6^.

The volume of saliva produced over a span of 24 hours ranges from 500 ml to 600 ml. The amount of saliva secreted in an unstimulated state is approximately 0.32 ml/minute; in a stimulated state, the secretion rate may reach 3–4 ml/minute ^3, 7^. Stimulation of the salivary glands can occur through olfactory stimulation; seeing and thinking about food; mechanical, chemical, or neuronal stimulation; and pain ^8, 9^. Mechanical stimulation occurs when an individual chews food or gum. Sweet, sour, salty, bitter, and spicy tastes provide chemical stimulation. Neuronal stimuli pass through the sympathetic and parasympathetic nerves. Pain due to inflammation, gingivitis, or an ill-fitting prosthesis could also stimulate salivary secretion. In addition, stress and psychological conditions may affect salivary secretion ^8^.

Bubble tea drinks are currently very popular throughout the world. The drink consists of a combination of tea and tapioca pearls. Pearl tapioca is a product made from sago starch ^10, 11^. The nutritional content of bubble tea drinks per 16 fl. oz. (472 ml) includes 317.5 calories, as much as 10.6 g of total fat, 56 g of carbohydrates, 36 g of sugar, and 1.8 g of protein ^12^. To date, studies on the effect of bubble tea drinks are scarce. Bubble tea could stimulate mastication via the chewing of tapioca pearls. Mechanical stimulation by chewing tapioca pearls and chemical stimulation by the tea in bubble tea drinks may improve the quality of saliva. Thus, bubble tea drinking may help prevent bacteria and viruses from entering the body.

Studies on saliva continue to be developed because samples may be collected noninvasively. Salivary CRP does not reflect systemic inflammatory conditions but may be influenced by the local oral environment ^13^. The objective of this study is to analyze the effect of chewing the tapioca pearls in bubble tea drinks on salivary CRP and Ca levels.

## Methods

The protocol of this study was registered through ClinicalTrials.gov, NCT04670341. This trial was registered after the trial start (17 ^th^ December 2020). 

### Subjects

This experiment had a pre/posttest test design that considered bubble tea drink (tea with tapioca pearls) consumption as the intervention and tea (without pearls) consumption as the control. The study protocol was given ethics committee approval (Ref. No. KE/FK/0866/EC/2020) and Amendment Approval from the Medical and Health Research Ethics Committee, Faculty of Medicine, Public Health and Nursing, Universitas Gadjah Mada–Dr. Sardjito General Hospital.

Sample size was calculated according to Lemeshow and David ^14^:


$$n\;=\;\frac{2{\left(Z_{1-\frac{\alpha }{2}}\;+\;Z_{1-\beta }\right)}^{2}\sigma^{2}}{{\left(\mu 1\;-\;\mu 2\right)}^{2}}$$

n = number of samples

Z _1-α/2 _= value in the standard normal distribution equal to the level of alpha α significance (1.96 for α = 0.05)

Z _1-β_ = value in the standard normal distribution equal to the desired power (1.28 for β = 0.01)

σ = standard deviation of the outcome

μ _1_ = mean outcome of the treatment group

μ _2_ = mean outcome of the control group

We calculated the sample size to be n = 14.33 ≈ 15 individuals.

Subject selection has a randomized controlled trial design. The inclusion criteria were any individuals aged from 18 to 25 years with a good Oral Hygiene Index-Simplified (OHI-S) score ^15^. Exclusion criteria were individuals taking medication, a history of dry mouth, smoking, and systemic disease. In addition, due to this study taking place during the Covid-19 pandemic, subjects were excluded if they did not have a negative Covid-19 test, as determined by a rapid test carried out before the study. Based on the literature, we specified *a priori* the potential covariates: infection within two weeks, systemic disease, medication, smoking ^16, 17^.

The subjects were students from the Faculty of Dentistry, Universitas Gadjah Mada, Yogyakarta, Indonesia. Method of selecting subjects was convenience sampling. Information about the study was sent to a select few prospective subjects and asked them to participate in this study. Students who agreed to participate filled in a form via Google Forms.. Data in the form included travel history, fever history, olfactory disturbance, out of breath, taste disturbances, and medical and dental history. All of the subjects who participated in this study provided written informed consent to participate.

According to health protocols, the body temperature of each subject was measured, and rapid testing for Covid-19 was conducted before OHI-S measurement and saliva collection. Rapid testing, OHI-S measurement, and saliva collection were performed at Korpagama Clinic, Universitas Gadjah Mada, Yogyakarta, Indonesia (Letter from the Faculty of Dentistry, Universitas Gadjah Mada to Korpagama Clinic No. 6901/UN1/FKG.1/Set.KG1/PT/2020).

### Saliva collection

In the first week of the experiment, the subjects were instructed to drink 100 ml of bubble tea over a span of 5 minutes once a day for 3 days (intervention week). In the second week, the same subjects drank tea without tapioca pearls (control week).

The participants were asked to drink a specific brand (Chatime) of tea. The students drank the tea in front of the researchers to ensure adherence to the protocol.

The subjects were instructed not to eat at least 60 minutes prior to saliva collection. Saliva was collected on the first day before bubble tea consumption (pretest) and on the third day after tea consumption (posttest) for both the intervention and control weeks; collection was conducted in the morning (09:00 a.m.–12.00 p.m.). The subjects were asked to stand, and saliva was collected from the oral cavity. Each subject was asked to spit into a saliva container for 1 minute. The saliva container was then closed tightly, sealed, and wiped clean with disinfectant tissue. The sample was placed in an aluminum bag that was then placed in a biohazard container. The saliva samples were stored in a freezer (−20°C) until CRP and calcium level measurements.

### Measurement of salivary C-reactive protein and calcium level

Salivary CRP and Ca level measurements were performed at the Parasitology Laboratorium, Faculty of Medicine, Public Health and Nursing, Universitas Gadjah Mada. Saliva samples were thawed completely, vortexed, and centrifuged at 1500× *g* for 15 minutes. Clear samples were pipetted into the appropriate dilution tube. Salivary CRP levels were measured using an ELISA kit (Item No. 1-2102, Salimetrics ^®^, State College, PA 16803, USA). Approximately 100 μl of the standard, control, and saliva samples were pipetted into the appropriate wells of a test plate. The plate was placed on a plate rotator at 500 rpm for 2 hours and room temperature for complete sample mixing. The plate was washed four times with 1× wash buffer. Exactly 100 μl of the conjugate solution was added to each well, and the plate was placed on the plate rotator once more at 500 rpm and room temperature. The plate was washed four times with 1× wash buffer. Exactly 100 μl of TMB substrate solution was added to each well. The plate was incubated in the dark at room temperature for 30 minutes, mixed for 5 minutes on the plate rotator at 500 rpm, and then added with 50 μl of stop solution. The absorbance of each well was read at 450 nm.

Ca level was assessed using a Ca test kit according to the manufacturer’s protocol (QUANTOFIX ^®^ Calcium, Catalog No. 91324, Macherey–Nagel GmbH & Co. KG, Germany).

### Data analysis

Data were analyzed using statistical measurement (SPSS v22, IBM). Shapiro Wilk and Levene tests were conducted to determine whether the data were normal and homogenous. Data were analyzed using the Kruskal–Wallis and Mann–Whitney tests to compare differences between the control and intervention weeks on the first and third days following tea consumption.

## Results

The CRP and Ca levels of the two groups are described in Table 1. CRP levels decreased whereas Ca levels increased on the third day in the control and intervention weeks. The results of the Shapiro–Wilk and Levene tests were less than 0.05; thus, the data were not normally or homogenously distributed. Therefore, the data were analyzed by the Kruskal–Wallis test.

CRP and Ca levels before and after drinking tea with and without bubble were significantly different (p < 0.05) in both weeks ( Table 3). This result indicates that bubble tea may have a significant effect on salivary CRP and Ca levels. CRP and Ca levels between groups were analyzed using the Mann–Whitney test ( Table 3 and Table 4).

**Table 1.** CRP and Ca levels in saliva of subjects consuming bubble tea with (intervention) and without (control) tapioca pearls (n=15).

Group		CRP (pg/ml)	Ca (mg/L)
Control week	Day 1	395.66 ± 185.14	33.00 ± 12.42
	Day 3	361.91 ± 170.93	45.67 ± 8.55
Intervention week	Day 1	247.06 ± 135.71	15.33 ± 11.78
	Day 3	90.97 ± 62.45	28.67 ± 21.91

Data are presented as mean ± SD. NB: the same subjects took part in the intervention and control weeks.

A comparison of salivary CRP levels ( Table 3) showed significant differences (p <0.05) between the first and third days in the intervention group, between the control and intervention groups the first day, and between the control and intervention groups on the third day. No significant difference in salivary CRP level was found between the first and third days in the control group (p >0.05). Table 4 compares Ca levels between the groups and shows significant differences between control the first and third day, control the third day and intervention first also third day, intervention the first and third day (p <0.05). There was no significant difference (p>0.05) between control the first day and intervention the first also third day.

## Discussion

The results of this study showed that bubble tea consumption with and without tapioca pearls decreases CRP levels in saliva but increases Ca levels on the third day in both the control and intervention weeks ( Table 1). The decrease in salivary CRP levels on the third day in the intervention group compared to the first day showed a significant difference ( Table 2). This finding indicates that bubble tea consumption and bubble chewing may reduce salivary CRP levels and increase salivary Ca levels.

**Table 2.** Kruskal–Wallis results of CRP and Ca levels.

	CRP level (pg/ml)	Ca level (mg/l)
Chi-squared df Asymp.Sig	23.393 3 0.000	19.283 3 0.000

**Table 3.** Mann–Whitney results of CRP levels in the saliva of the control and intervention groups.

	Control first day	Control third day	Intervention first day	Intervention third day
Control first day	-	0.648	0.021*	0.048*
Control third day		-	0.461	0.000*
Intervention first day			-	0.004*
Intervention third day				-

**Table 4.** Mann–Whitney results of Ca levels in the saliva of the control and intervention groups.

	Control first day	Control third day	Intervention first day	Intervention third day
Control first day	-	0.041*	0.174	0.744
Control third day		-	0.000*	0.001*
Intervention first day			-	0.015*
Intervention third day				-

Table 3 revealed no significant difference in salivary CRP level between the first and third days in the control group. By contrast, the intervention group revealed a significant decrease in salivary CRP levels between the first and third days. This result is supported by Pay and Shaw ^13^, who found that salivary CRP may be influenced by the oral environment. Chewing bubble tea containing tapioca pearls could stimulate saliva secretion mechanically. Masticatory stimulation by chewing tapioca pearls may stimulate the salivary gland to produce more saliva. The stimulated saliva may also increase organic, inorganic, and salivary protein such as mucin, α-amylase, lysozyme, and peroxidase. The decrease in CRP level on the third day in the intervention group may be due to increased salivary secretion after bubble chewing. Chewing is one of factors that can stimulate salivary secretion ^2^. Increased salivary secretion could enhance the function of saliva as a lubricant and antimicrobe activity that could reduce bacteria and viruses entering the oral cavity. Decreasing bacteria and viruses in the oral cavity will reduce microbes that cause inflammation, therefore reducing CRP level ^18^.

Salivary CRP levels in the control week after drinking tea without bubbles on the third day decreased compared with those on the first day ( Table 1), but the difference noted was not statistically insignificant ( Table 3). We controlled for the effect of inflammation on the gingiva by only including subjects that had an OHI-S criteria good category. The decrease in salivary CRP level on the third day in the intervention week may be attributed to the various components of tea, which have antibacterial and antiviral functions. Epigallocatechin gallate is the most polyphenolic catechin found in tea and may be a potential treatment option against several viruses ^19^. This result supports a previous finding that consumption of green tea could enhance the antibacterial capacity of saliva ^20^. Tea polyphenols may also have antiviral functions. Mhatre *et al.* ^19^ found that tea is a potential candidate for the prophylaxis and treatment of COVID-19.

Calcium levels increased significantly on the third day compared with that on the first day in the control and intervention weeks ( Table 1 and Table 4). Salivary flow rate may be related to salivary Ca so that the increase saliva secretion is in line with an increase in salivary Ca ^21^. Salivary Ca have role to maintain the integrity of intraoral mineralization. In our study, all subjects were aged 21–22 years and had good OHI-S scores. Increased Ca levels after consumption of tea with and without bubbles may indicate improvements in saliva quality to maintain oral homeostasis. Our result supports the findings of a previous study that found that high salivary levels are correlated with good dental health in young adults ^22^.

## Conclusion

Based on our data, we conclude that consuming bubble tea drinks could improve the quality of saliva by decreasing salivary CRP and increasing Ca levels.

## Data availability

### Underlying data

Figshare: Raw data subject-CRP-Calcium, https://doi.org/10.6084/m9.figshare.13139711.v1 ^23^.

### Reporting guidelines

Figshare: CONSORT checklist and flow diagram for ‘Quality improvement of saliva by chewing tapioca pearls in bubble tea drinks: a randomized experimental trial’, https://doi.org/10.6084/m9.figshare.13585241.v1 ^24^.

Data are available under the terms of the Creative Commons Attribution 4.0 International license (CC-BY 4.0).
