# Supplementary material for: Quality improvement of saliva by chewing tapioca pearls in bubble tea drinks: a randomized experimental trial: (Study on salivary C-reactive protein (CRP) and calcium (Ca) levels)
Source: F1000Res. 2022 Mar 7;10:56. Originally published 2021 Feb 1. [Version 2] doi: 10.12688/f1000research.28028.2 (PMC8938629; doi:10.12688/f1000research.28028.2)
Supplement: Peer review history

# Peer review: Reviewer response for version 2

**DOI:** 10.5256/f1000research.120355.r126439

**Published:** 2022-04-19

**Authors:** Paulo H. Braz-Silva

**Affiliations:** 1 Department of Stomatology, School of Dentistry, University of São Paulo (USP), São Paulo, Brazil

As stated in my last report, the conclusions of the study are not supported by the data.

Is the work clearly and accurately presented and does it cite the current literature?

No

If applicable, is the statistical analysis and its interpretation appropriate?

I cannot comment. A qualified statistician is required.

Are all the source data underlying the results available to ensure full reproducibility?

Yes

Is the study design appropriate and is the work technically sound?

No

Are the conclusions drawn adequately supported by the results?

No

Are sufficient details of methods and analysis provided to allow replication by others?

No

Reviewer Expertise:

Oral and Maxillofacial Pathology; Virology; Salivary biomarkers

I confirm that I have read this submission and believe that I have an appropriate level of expertise to state that I do not consider it to be of an acceptable scientific standard, for reasons outlined above.

# Peer review: Reviewer response for version 2

**DOI:** 10.5256/f1000research.120355.r126438

**Published:** 2022-03-21

**Authors:** Muhammad Sohail Zafar

**Affiliations:** 1 Department of Restorative Dentistry, College of Dentistry, Taibah University, Medina, Saudi Arabia

The manuscript is well presented.

Is the work clearly and accurately presented and does it cite the current literature?

Yes

If applicable, is the statistical analysis and its interpretation appropriate?

Yes

Are all the source data underlying the results available to ensure full reproducibility?

Yes

Is the study design appropriate and is the work technically sound?

Yes

Are the conclusions drawn adequately supported by the results?

Yes

Are sufficient details of methods and analysis provided to allow replication by others?

Yes

Reviewer Expertise:

Dentistry

I confirm that I have read this submission and believe that I have an appropriate level of expertise to confirm that it is of an acceptable scientific standard.

# Peer review: Reviewer response for version 1

**DOI:** 10.5256/f1000research.31000.r100002

**Published:** 2022-01-11

**Authors:** Paulo H. Braz-Silva

**Affiliations:** 1 Department of Stomatology, School of Dentistry, University of São Paulo (USP), São Paulo, Brazil

Dear authors, 

 Thank you for the possibility to review this interesting manuscript. Although the topic is interesting, I have some major concerns about your study design. 

1. Your study population is poorly described. It is mandatory to state at least the oral health condition of the participants. 
2. Why did you choose the biomarkers C-reative protein (CRP) and calcium (Ca) levels to analyze the saliva quality? 
3. Why did you choose bubble tea drink as an intervention in your study? A chewing gum could have the same role? 
4. The discussion section is extremely superficial. 
5. Your conclusion is not supported by your results. 

Is the work clearly and accurately presented and does it cite the current literature?

No

If applicable, is the statistical analysis and its interpretation appropriate?

I cannot comment. A qualified statistician is required.

Are all the source data underlying the results available to ensure full reproducibility?

Yes

Is the study design appropriate and is the work technically sound?

No

Are the conclusions drawn adequately supported by the results?

No

Are sufficient details of methods and analysis provided to allow replication by others?

No

Reviewer Expertise:

Oral and Maxillofacial Pathology; Virology; Salivary biomarkers

I confirm that I have read this submission and believe that I have an appropriate level of expertise to state that I do not consider it to be of an acceptable scientific standard, for reasons outlined above.

# Peer review: Reviewer response for version 1

**DOI:** 10.5256/f1000research.31000.r100368

**Published:** 2022-01-11

**Authors:** Muhammad Sohail Zafar

**Affiliations:** 1 Department of Restorative Dentistry, College of Dentistry, Taibah University, Medina, Saudi Arabia

Reviewing the manuscript; “Quality improvement of saliva by chewing tapioca pearls in bubble tea drinks: a randomized experimental trial” there is need of some improvement before it can be indexed. Authors may consider the following points to improve this manuscript further.

- Please clarify, what method was used for randomisation and blinding process? If not, then it should not be called a randomised clinical trial, instead it should be called controlled clinical trial.
- Please clarify, what method was used for allocation concealment?
- Authors should write COVID-19 instead of Covid-19.
- Please carefully check the use of abbreviations throughout the manuscript, abstract, figures and tables.
- For randomised controlled clinical trial, it is better to use CONSORT checklist.

Is the work clearly and accurately presented and does it cite the current literature?

Yes

If applicable, is the statistical analysis and its interpretation appropriate?

Yes

Are all the source data underlying the results available to ensure full reproducibility?

Yes

Is the study design appropriate and is the work technically sound?

Yes

Are the conclusions drawn adequately supported by the results?

Yes

Are sufficient details of methods and analysis provided to allow replication by others?

Yes

Reviewer Expertise:

Dentistry

I confirm that I have read this submission and believe that I have an appropriate level of expertise to confirm that it is of an acceptable scientific standard, however I have significant reservations, as outlined above.

# Response

**Published:** 2022-01-15

**Authors:** Juni Handajani

**Affiliations:** Universitas Gadjah Mada, Indonesia

Thank you for your review and suggestions. I've revised the manuscript:

 1. We added an explanation of the randomization and blinding process in the method.

 2. We added allocation concealment in the method

 3. We have revised the writing of COVID-19

 4. We have used the CONSORT checklist http://www.doi.org/10.6084/m9.figshare.13585241.v1

# Peer review: Reviewer response for version 1

**DOI:** 10.5256/f1000research.31000.r78557

**Published:** 2021-02-04

**Authors:** Hendrik Setia Budi

**Affiliations:** 1 Department of Oral Biology, Universitas Airlangga, Surabaya, Indonesia

The article is well written, but needs clarification on a few statements below:

1. In the title, I suggest mentioning the CRP and CA level here, because the title should be clear.
2. In the introduction, please explain what is the means of saliva quality? The saliva increased or salivary protein increased as antibodies to the bacteria and viruses?
3. In the result, why didn't you show the saliva volume in the tables so that we can clearly see the mechanism in it. I mean not statistical table only.
4. In the conclusion, why is the use of tapioca pearl not mentioned in the conclusion? Please check in the discussion.
5. Overall the paper is interesting to know furthermore.

Is the work clearly and accurately presented and does it cite the current literature?

Yes

If applicable, is the statistical analysis and its interpretation appropriate?

Yes

Are all the source data underlying the results available to ensure full reproducibility?

Partly

Is the study design appropriate and is the work technically sound?

Yes

Are the conclusions drawn adequately supported by the results?

Yes

Are sufficient details of methods and analysis provided to allow replication by others?

Yes

Reviewer Expertise:

oral biology, dental pharmacology, toxicology, cell culture, and herbal medicine

I confirm that I have read this submission and believe that I have an appropriate level of expertise to confirm that it is of an acceptable scientific standard.

# Response

**Published:** 2022-01-15

**Authors:** Juni Handajani

**Affiliations:** Universitas Gadjah Mada, Indonesia

Thank you for your review and suggestions. I've added in the revised manuscript:

 1. Sub title (Study on salivary C-reactive protein (CRP) and calcium (Ca) levels)

 2. Explanation of saliva quality in the introduction

 3. Data of saliva volume in table 1

 4. Tapioca pearl in conclusion
